# An enterprise composite blockchain construction method for business environment

**DOI:** 10.1371/journal.pone.0299162

**Published:** 2024-03-01

**Authors:** Su Li, Junlu Wang, Baoyan Song

**Affiliations:** School of Information, Liaoning University, Shenyang, Liaoning, China; ICFAI Foundation for Higher Education Faculty of Science and Technology, INDIA

## Abstract

In order to foster a modern economic system and facilitate high-quality economic development, it is crucial to establish a conducive business environment. Undoubtedly, the evaluation of the business environment for enterprises constitutes a prominent area of research. Nevertheless, ensuring the authenticity and security of the raw data sources provided by participating enterprises poses a challenge, thereby compromising the accuracy of the evaluation. To tackle this issue, an enterprise composite blockchain construction method for business environment is proposed in this paper, which stores the raw data of enterprises by the means of hybrid on-chain and off-chain. Initially, the enhanced hash function SHA256 is introduced to encrypt the raw data of enterprises. The encrypted data is subsequently stored in an off-chain Level DB database, which is based on non-volatile memory. This approach effectively alleviates the burden on communication and storage. Secondly, a composite storage strategy on-chain is adopted: the key values from the Level DB are stored in the DAG-based Conflux public blockchain, while the enterprise state data is stored in the consortium blockchain, so as to provide trusted evidence of business environment evaluation data. Finally, it is demonstrated through a large number of experimental comparisons that the enterprise composite blockchain construction method proposed in this paper exhibits better read and write performance, lower storage efficiency and storage overhead, and outperforms both the before-improved Level DB database and existing blockchain storage models.

## 1 Introduction

The business environment covers the sum of the time and cost of complying with all aspects of policies and regulations from the start-up, operation to the end of the enterprise, which includes Starting a Enterprise, Dealing with Construction Permits, Getting Electricity and Credit, Registering Property, Protecting Minority Investors, Enforcing Contracts, Trading across Borders, Paying Taxes, Resolving Insolvency, as well as the social, economic, political and legal that impact enterprise activities. In summary, it is a systemic project that involves various policy reforms in multiple areas, including but not limited to, opening to the outside world and enhancing communication horizontally and vertically, and so forth [1]. For a city, a favorable business environment will accelerate the economic development and assist it to grow stronger [2]. As such, it is imperative to store the data generated from the whole process of enterprise operation, as proof for the business environment evaluation. Note that, blockchain, with decentralized, tamper-proof and traceable features [3–5], has substantial advantages in guaranteeing data security and integrity, which coincides with the urgent necessity to enhance data authenticity and security for business environment evaluation.

Unlike traditional databases, blockchain makes it almost impossible to delete or manipulate recorded information through the use of cryptographic methods [6], and it has the unique features of decentralization, traceability, trustworthiness, open sharing, independence, stability, accuracy, and efficiency [7], which fuel productivity gains, time and money savings, data security assurance, and rapid access to information. As a result, blockchain technology has been widely adopted in various fields such as finance, public services, Internet of Things, cyber security, supply chain, and so forth [8–12]. Nevertheless, the majority of traditional blockchains ignore diverse range of data types, large data quantity, and strong correlations between data during storage, resulting in the existing blockchain storage structure deficiencies in throughput, transaction confirmation speed and scalability, and making it will fail on short-time and high-concurrent data scenarios. At the same time, existing efforts are concentrated in the fields of finance, education, Internet of Things and logistics, but the research on "blockchain + enterprise business environment evaluation" is extremely rare. And the traditional method does not take into account the security and integrity of the raw data of enterprises when evaluating the enterprise business environment.

To this end, in order to better evaluate and analysis enterprise business environment, this paper delves deep into the storage method of enterprise raw data of business environment and proposes an enterprise composite blockchain construction method, which introduces Non-Volatile Memory (NVM)-based Level DB [13] and Directed Acyclic Graph (DAG) [14]-based Conflux [15]. In chronological order, the raw data of the enterprise is stored utilizing a classified and efficient manner, which is guaranteed to be tamper-proof, traceable, and mutually recognized by multiple parties. The method proposed in this paper can also be applied in various areas, including supply chain management, financial services, identity management, and the Internet of Things (IoT). In supply chain management, this paper’s method enables businesses to integrate multiple systems onto one platform, improving coordination and efficiency. In financial services, it eliminates intermediaries, reduces costs, and ensures secure transactions. For identity management, it provides a trusted system for secure verification. In IoT, it enables secure communication and ensures data integrity and privacy.

The main contributions of this paper are as follows:

1. To the best of our knowledge, to assess the business environment of enterprises, this paper firstly applies the blockchain technology to the raw data storage process for enterprise business environment. Focusing on the security and efficiency of data storage, an enterprise composite blockchain construction method for business environment is devised to ensure the tamper-proof and traceability of data.
2. To ensure the security demand of enterprise raw data tamper-proof, this paper puts forward an improved hash function SHA256 algorithm. In essence, it increases the number of hashing operations in the data block and the complexity of the algorithm logic and compression function, so as to enhance the collision resistance of the operation and the avalanche effect of the Hash results, thus further enhancing the security of the raw data.
3. In view of the characteristics of enterprise business environment data such as diverse range of data types, large data quantity, and strong correlations, this paper presents a storage mode that synergizes on-chain and off-chain to further degrade the communication and storage burden on the blockchain system. For off-chain storage, an NVM-based off-chain Level DB storage model is proposed. In Level DB, the Hash value encrypted by the improved SHA256 algorithm is as the Key value, and the Value value is the enterprise raw data.
4. For on-chain, disregard of data association relationships, vanilla blockchains result in slow transaction confirmation and low throughput. In response to the limitations, this paper proposes a composite blockchain construction method, where the public blockchain adopts the DAG-based Conflux to store the Key values in the off-chain Level DB database, and the consortium blockchain stores the state data of enterprises, i.e., the correlation relationship between enterprises. The novel storage mode further improves the storage efficiency and provides support for the evaluation of enterprise business environment.

The subsequent sections of this paper are organized as follows. Second 2 is mostly concerned about related work. In Section 3 and 4, the enterprise composite blockchain construction approach for the business environment proposed in the research is discussed in detail. Section 5 includes several experiments and analyses to demonstrate the superior performance of the enterprise composite blockchain construction approach. We form a conclusion and recommend further research in Section 6.

## 2 Related work

Until now, the scholars at home and abroad have carried out a surge of extensive and thorough research on Level DB optimization and blockchain construction, and have achieved significant advancements.

In regard to Level DB optimization. Dai [16] et al. proposed the Bourbon method, which uses greedy piecewise linear regression to learn the key distribution and achieve fast lookup with minimal computation, but the Bourbon method complicated garbage collection. FlatStore [17], a PM-based key-value storage engine, decoupled the roles of KV storage into persistent log structures and volatile indexes, and integrated two technologies to provide low-latency and high-throughput performance. However, this storage engine required excessive CPU resources due to the compressed log format. Zhang [18] et al. proposed ChameleonDB, which employed an LSM-tree structure to efficiently admit writes with low write amplification, while it used an in-DRAM hash table to bypass multiple levels of the LSM-tree for fast reads. However, ChameleonDB was not suitable for distributed environments. Yang [19] et al. proposed a design method based on nonvolatile memory and machine learning. It utilized persistent variable memory tables on the NVM, which reduced access latency to some extent, but led to more severe write stalls.

In the realm of blockchain construction. Zamyatin et al [20] devised Inclusive blockchain, which extends Nakamoto consensus and GHOST rules to DAG and designed a framework to include off-chain transactions. Sompolinsky et al [21] proposed the PHANTOM platform, where participating nodes firstly found an approximate k-cluster solution for their local block DAG to prune potentially malicious blocks. Subsequently, the remaining blocks were topologically sorted to obtain the final total block order. Nevertheless, both Inclusive blockchain and PHANTOM are susceptible to attacks when the block generation rate is high. Das et al [22] proposed to build the Bitcoin-NG blockchain, which improves the throughput by periodically electing a leader and allowing it to specify the full order of transactions over a period of time, but failed to improve the confirmation time of transactions. Gazsi et al [23] proposed the Vault blockchain, where Vault employed the sharding technique to construct the blockchain to decrease the storage cost while balancing network bandwidth cost. Although the combined throughput of all the shards was high, the throughput of inter-shard transactions was still limited.

### 2.1 Comparison

Due to the deficiencies such as slow data merging on disks, the need to reorder write-to-disk data, previous work such as Bourbon, FlatStore, ChameleonDB and other methods will lead to writing stall, writing amplification, and poor read performance during Level DB optimization. In addition, traditional blockchains such as Inclusive, PHANTOM, Bitcoin-NG and other storage structures fail to take into account the storage burden in the case of diverse data types and large data quantity, resulting in low throughput, slow confirmation speed and poor scalability. As such, this paper integrates Level DB optimization and blockchain storage efficiency and overhead, and proposes an enterprise composite blockchain construction method for business environment, which can improve aforementioned shortcomings and significantly improve read and write performance, storage efficiency and reduce storage overhead.

## 3 Enterprise raw data off-chain storage

**Fig 1:**
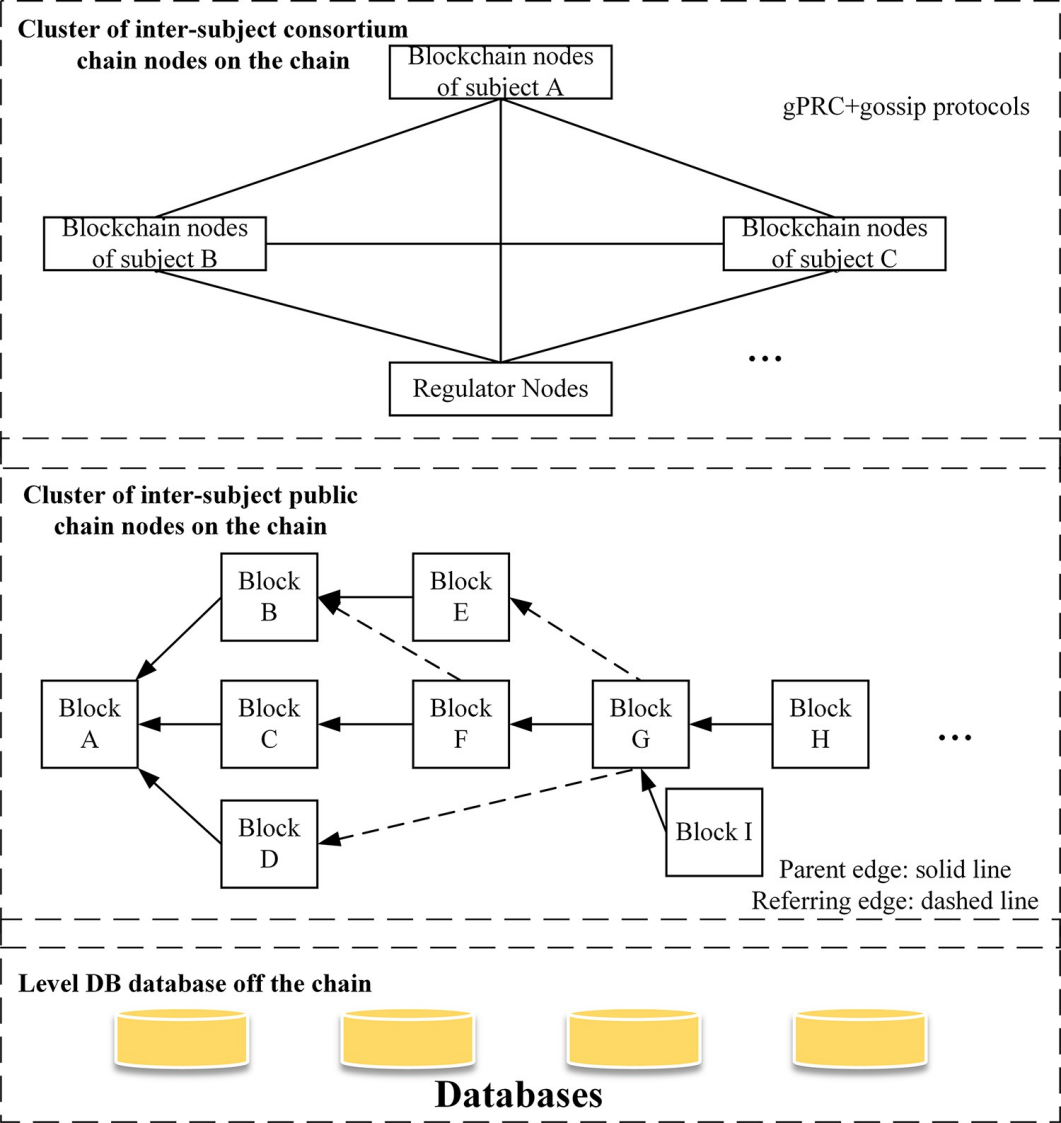
Enterprise composite blockchain architecture diagram.

As shown in Fig 1, the overall architecture of the enterprise composite blockchain comprises Level DB database, public blockchain and consortium blockchain. The Level DB database stores the raw data of the enterprise, where Value is the raw data, and Key is the Hash value encrypted by the improved SHA256 algorithm. The Public blockchain, responsible for transactions, stores the Key value corresponding to the raw data in the enterprise Level DB database, while the consortium blockchain stores the state data of the enterprise. Through the abovementioned architecture, the data collaboration between on-chain and off-chain is realized. The on-chain realizes the capacities of computing and storage through the off-chain, and the off-chain and on-chain docking realizes the sharing of heterogeneous information.

When storing, managing, and searching the vast data generated throughout the enterprise process from start-up, operation to closure in an off-chain, persistent and trustworthy manner, vanilla SQL databases may not be sufficient because of their limitations in performance and scalability. Instead, Level DB database exhibits excellent concurrent read and write performance, and can achieve horizontal and vertical expansion of data storage to meet availability and partition tolerance while ensuring ultimate consistency. Therefore, Level DB database with high reliability and scalability can meet the aforesaid requirements. When data is on the chain, the enterprise composite blockchain storage architecture is built, and the DAG-based public blockchain is utilized to store the Key values corresponding to the enterprise raw data in the Level DB database off the chain to achieve high throughput and fast confirmation. The consortium blockchain stores enterprise state data, enabling quick retrieval of corresponding enterprise state information for business environment evaluation.

### 3.1 Enterprise raw data encryption calculation

Prior to storing the raw data into the off-chain Level DB database, the hash function SHA256 algorithm is employed to encrypt the raw data of the enterprise. When queried, it enables verification of whether the data has been tampered with. SHA256 converts an arbitrarily long message into a shorter, fixed-length message digest, which is used as the Key value in the key-value pair, and the Value corresponds to the raw data, which is stored in Level DB database, meanwhile the Key value stores to the Conflux public blockchain in the enterprise composite blockchain. To ensure the security demand of enterprise raw data tamper-proof, this paper puts forward an improved hash function SHA256 algorithm to encrypting raw data. In essence, it increases the number of hashing operations in the data block and the complexity of the algorithm logic and compression function, so as to enhance the collision resistance of the operation and the avalanche effect of the Hash results, thus enhancing further security.

SHA256 algorithm adopts six logical functions and a set of constant *K*_*t*_, the input is a 512bit message block *x*_*i*_, and then divided into 16 groups of 32bit byte *M*_0_,*M*_1_,…,*M*_15_, the output is a 256bit message digest [24], SHA256 algorithm process is as follows.

1. Initialization
$$\begin{array}{l} H_{0}^{\left(0\right)}=0x6a09e667,H_{1}^{\left(0\right)}=0xbb67ae85,\\ H_{2}^{\left(0\right)}=0x3c6ef372,H_{3}^{\left(0\right)}=0xa54ff53a,\\ H_{4}^{\left(0\right)}=0x510e527f,H_{5}^{\left(0\right)}=0x9b05688c,\\ H_{6}^{\left(0\right)}=0x1f83d9ab,H_{7}^{\left(0\right)}=0x5be0cd19 \end{array}$$ (1)
2. Prepare message list *W*_*t*_
$$W_{t}=M_{t}^{\left(i\right)}(0\leq t\leq 15)$$ (2)
$$W_{t}=\sigma_{1}^{\left\{256\right\}}(W_{t-2})+W_{t-7}+\sigma_{0}^{\left\{256\right\}}(W_{t-15})+W_{t-16}(16\leq t\leq 163)$$ (3)
  where the logical function is calculated as follows
$$\sigma_{0}^{\left\{256\right\}}=ROTR^{7}(x)⊕POTR^{18}(x)⊕ROTR^{3}(x)$$ (4)
$$\sigma_{1}^{\left\{256\right\}}=ROTR^{17}(x)⊕POTR^{19}(x)⊕ROTR^{10}(x)$$ (5)
3. Initialize eight working variables *A*、*B*、*C*、*D*、*E*、*F*、*G*、*H*_0_ according to $H_{0}^{\left(0\right)}∼H_{7}^{\left(0\right)}$, and then assign values to them using the intermediate results of each round of Hash values.
4. When 0≤*t*≤63, carry out the compression function
$$T_{1}=h+\sum_{1}^{\left\{256\right\}}\left(e\right)+Ch(e,f,g)+K_{t}^{\left(256\right)}+W_{t}$$ (6)
$$T_{2}=\sum_{0}^{\left\{256\right\}}\left(a\right)+M_{aj}(a,b,c)$$ (7)
$$h=g,g=f,f=e,e=d+T_{1},d=c,c=b,b=a,a=T_{1}+T_{2}$$ (8)
  where the logical function is calculated as follows
Ch(x,y,z)=(x∧y)⊕(¬x∧z) (9)
$$\sum_{1}^{\left\{256\right\}}\left(x\right)=ROTR^{6}(x)⊕POTR^{11}(x)⊕ROTR^{25}(x)$$ (10)
$$M_{ai}(x,y,z)=(x\wedge y)⊕(x\wedge z)⊕(y\wedge z)$$ (11)
$$\sum_{0}^{\left\{256\right\}}\left(x)=ROTR^{2}(x\right)⊕POTR^{13}(x)⊕ROTR^{22}(x)$$ (12)
5. Calculate the middle hash for each grouping
$$\begin{array}{l} H_{0}^{\left(i\right)}=a+H_{0}^{\left(i-1\right)},H_{1}^{\left(i\right)}=b+H_{1}^{\left(i-1\right)},\\ H_{2}^{\left(i\right)}=c+H_{2}^{\left(i-1\right)},H_{3}^{\left(i\right)}=d+H_{3}^{\left(i-1\right)},\\ H_{4}^{\left(i\right)}=e+H_{4}^{\left(i-1\right)},H_{5}^{\left(i\right)}=f+H_{5}^{\left(i-1\right)},\\ H_{6}^{\left(i\right)}=g+H_{6}^{\left(i-1\right)},H_{7}^{\left(i\right)}=h+H_{7}^{\left(i-1\right)} \end{array}$$ (13)
  where *i* is the *i*th group of the message, and after all groups are processed, the Hash value of 256bit is output.

In this paper, the improved SHA256 algorithm firstly adds 16 operations to each 512-bit data block hash operation to increase nonlinear diffusion, thus ensuring that each bit of the message can affect more bits. Moreover, the complexity of the SHA256 algorithm logic and compression function is increased to accelerate the differential diffusion of messages and make the recursive process more "random", which thus eliminates the dependency condition of local collision. The pipeline of improved SHA256 algorithm is depicted as follows.

(1) For message block *M*^(*i*)^,*i* = 1,2,…,*N*, perform the following loop.
  1) Prepare message list *W*_*t*_:
$$W_{t}=M_{t}^{\left(i\right)}(0\leq t\leq 15)$$ (14)
$$W_{t}=W_{t-4}+\sigma_{0}(W_{t-9})+\sigma_{1}(W_{t-12})+W_{t-16}(16\leq t\leq 79)$$ (15)
  2) Working variable initialization:
$$\begin{array}{l} A=H_{0}^{\left(i-1\right)},B=H_{1}^{\left(i-1\right)},C=H_{2}^{\left(i-1\right)},D=H_{3}^{\left(i-1\right)}\\ E=H_{4}^{\left(i-1\right)},F=H_{5}^{\left(i-1\right)},G=H_{6}^{\left(i-1\right)},H=H_{7}^{\left(i-1\right)} \end{array}$$ (16)
    where *A*~*H* denotes 8 working variables of length 32bit; $H_{0}^{\left(i-1\right)}∼H_{7}^{\left(i-1\right)}$ denotes the Hash value in the previous round of calculation, when *i* = 1, $H_{0}^{\left(0\right)}∼H_{7}^{\left(0\right)}$ are constant initial values, which can be chosen randomly, with reference to the unimproved SHA256 algorithm, set as
$$\begin{array}{l} H_{0}^{\left(0\right)}=0x6a09e667,H_{1}^{\left(0\right)}=0xbb67ae85,\\ H_{2}^{\left(0\right)}=0x3c6ef372,H_{3}^{\left(0\right)}=0xa54ff53a,\\ H_{4}^{\left(0\right)}=0x510e527f,H_{5}^{\left(0\right)}=0x9b05688c,\\ H_{6}^{\left(0\right)}=0x1f83d9ab,H_{7}^{\left(0\right)}=0x5be0cd19 \end{array}$$ (17)
(2) When 0≤*t*≤79, carry out the following compression function

$$T_{1}=h+\sum_{0}(e)+Ch(e,f,g)+K_{t}+W_{t}$$ (18)

$$T_{2}=\sum_{1}\left(a)+M_{aj}(a,b,c\right)$$ (19)

$$\begin{array}{l} h=g+T_{1},g=f+T_{2},f=e+T_{1},e=d+T_{2},\\ d=c+T_{1},c=b+T_{2},b=a+T_{1},a=T_{1}+T_{2} \end{array}$$ (20)

where *T*_1_,*T*_2_ is the intermediate variable and the compression function uses a sequence of 80 32-bit words of the $K_{t}^{\left\{256\right\}}\;(0\leq t\leq 79)$ constant, the first 64 32-bit byte are given in Ref. [25] and the latter 16 32-bit byte are defined as follows

$$\begin{array}{llll} 0x328a3f95 & 0x61375461 & 0xa5c0fbef & 0xf9b5dca7\\ 0x2953c25e & 0x59f211f3 & 0x623f82a6 & 0xad1c5ed4\\ 0xd507ab93 & 0x12634b05 & 0x223175bf & 0x750c7ac5\\ 0x76ba5d34 & 0x84dec1fd & 0x7bac06a4 & 0xc17bf162 \end{array}$$ (21)

The six logical functions employed by the improved SHA256 algorithm are all based on 32-bit byte (e.g. *x*,*y*,*z*) for operation, and the result of each logical function is a new 32-bit byte, the specific definitions are as follows

$$\begin{array}{l} \sigma_{0}(x)=ROTR^{8}(x)⊕ROTR^{18}(x)⊕SHR^{5}(x)\\ \sigma_{1}(x)=ROTR^{10}(x)⊕ROTR^{23}(x)⊕SHR^{16}(x)\\ \sum_{0}(x)=ROTR^{6}(x)⊕ROTR^{14}(x)⊕ROTR^{21}(x)\\ \sum_{1}(x)=ROTR^{3}(x)⊕ROTR^{11}(x)⊕ROTR^{27}(x)\\ Ch(x,y,z)=(x\wedge y)⊕(\neg x\wedge z)⊕(\neg y\wedge \neg z)\\ M_{aj}(x,y,z)=(x\wedge y)⊕(x\wedge z)⊕(y\wedge z) \end{array}$$ (22)

**Fig 2:**
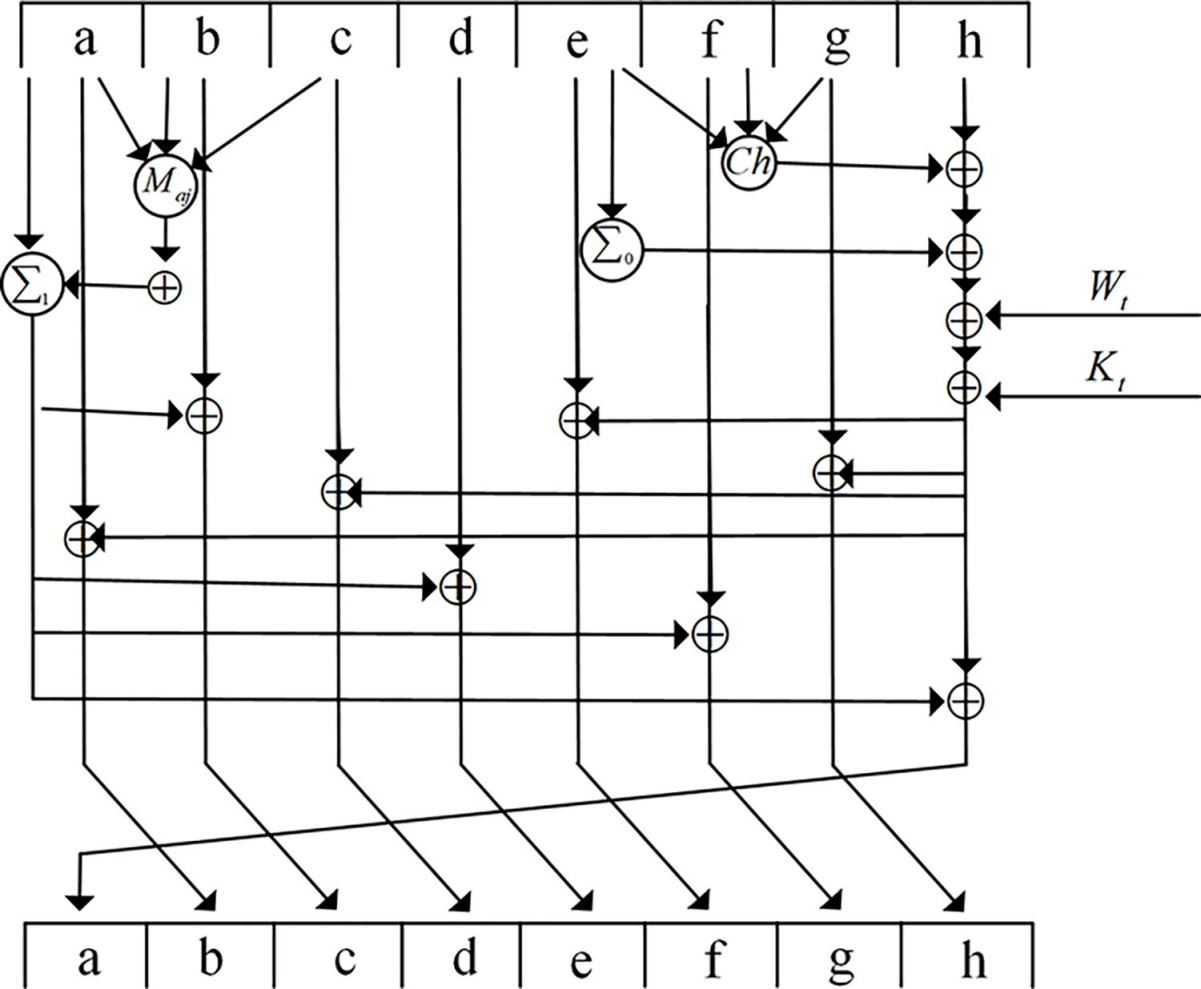
Improved SHA256 algorithm operation diagram.

The loop operation of each group is shown in Fig 2.

The aforementioned improvement measures enhance unidirectionality, nonlinearity, pseudo-randomness, collision resistance and avalanche effect of SHA256. The improved SHA256 algorithm is employed to encrypt the raw data of the enterprise and generate the corresponding Hash value as the Key value in the off-chain Level DB database for subsequent storage operations.

### 3.2 NVM-based off-chain Level DB storage model construction

**Fig 3:**
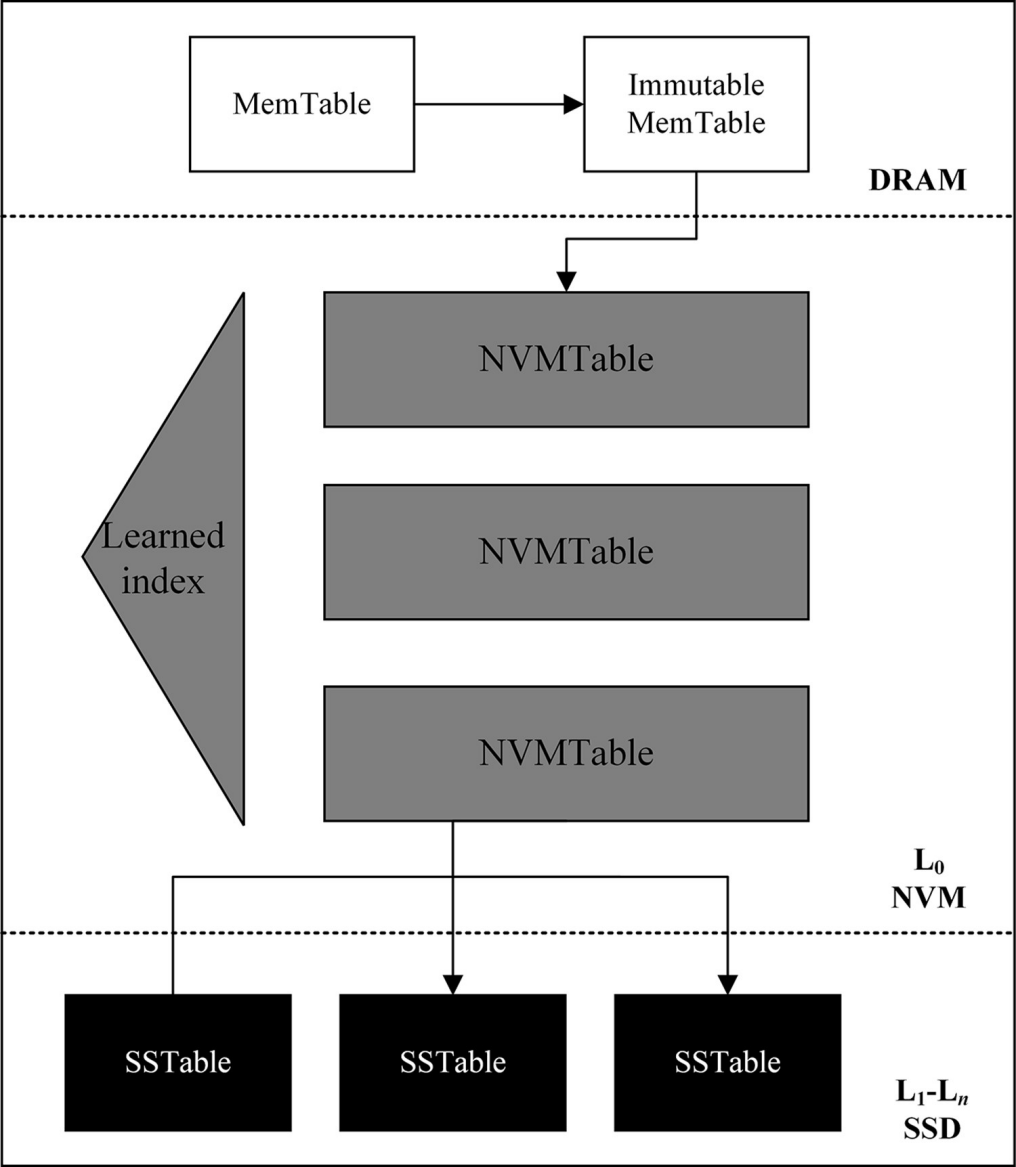
Off-chain Level DB storage model architecture diagram.

After the encryption calculation of the improved hash function SHA256 algorithm, the encrypted Hash value of the raw enterprise data is used as the Key value and the raw data is used as the Value value, which are correspondingly deposited into the off-chain Key-Value type database Level DB. Level DB is a Key-Value non-relational database storage system based on Log-structured Merge Tree (LSM-tree) architecture, which is widely used in many fields due to its advantages of efficient writing and minimal space usage. However, the LSM-tree exhibit limitations such as writing stall, writing amplification and unfavorable reading. Therefore, this paper proposes an NVM-based LSM-tree storage model, and the off-chain Level DB storage model architecture is shown in Fig 3.

As shown in Fig 3, Layer *L*_0_ from LSM-tree architecture is placed on NVM, and layers *L*_1_ to *L*_*n*_ are stored on Solid State Disk (SSD) to address writing latency when data are merged. And a learning index is introduced to the files in *L*_0_ to make the files in layer *L*_0_ orderly and decrease read latency, and improve data searching speed.

**Fig 4:**
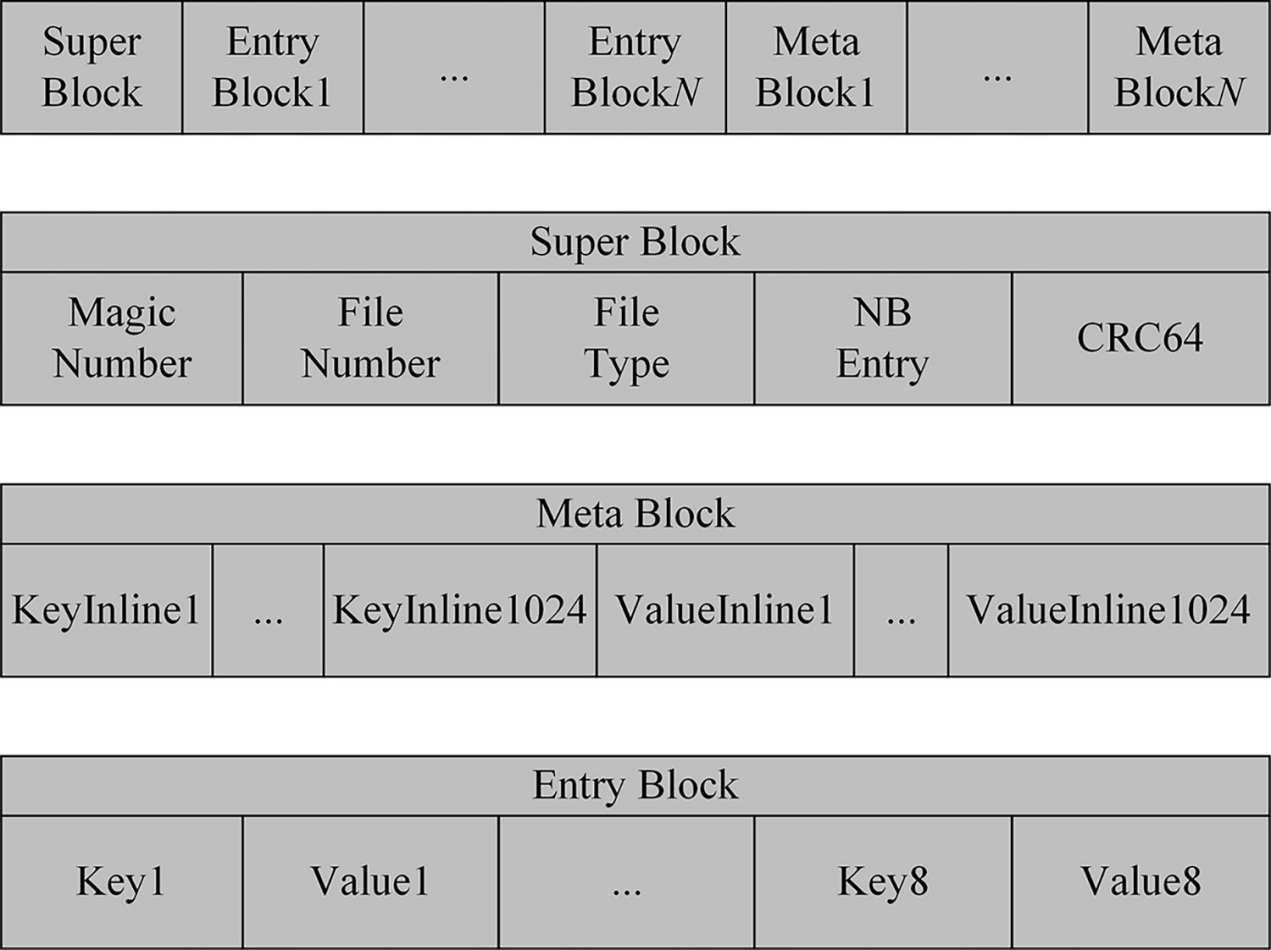
NVMTable format diagram.

Fig 4 showcases the organization form of the data in *L*_0_ on NVM. NVMTable is divided into three types of blocks based on storage content, namely Super Block, Meta Block, and Entry Block.

As shown in Fig 4, Super Block is the first Block of each NVMTable, which is responsible for storing the meta information of the file and contains the attribute fields as shown in Table 1; Entry Block is responsible for storing key-value pairs or pointers; Meta Block is responsible for storing the bitmap information of 1024 key-value pairs, which marks whether a key or value is saved in the Entry Block as the real data or a pointer.

**Table 1:** Attribute fields contained in Super Block.

Fields	Meanings
Magic number	Determine if the current file format is NVMTable
File number	File number
File type	File type
NB entry	Number of key-value pairs in the file
CRC64	File check code

When Minor Compaction occurs that involves writing Immutable MemTable to the disk *L*_0_ and converting it to SSTable, the data needs to be written to NVMTable, and meanwhile, files NVMTable and Container will be created. In the process of writing data, firstly, determine whether the lengths of the Key and Value data is shorter than 32 B. If so, it is an inline data and write it directly to the Entry Block; otherwise, write the corresponding data pointer in the file Container. Secondly, while writing data to the Entry Block, Meta Block is created in memory until NVMTable no longer writes data. Subsequently, the Meta Block is appended to the end of the file. Finally, the Super Block in the head of the file is updated to complete the write operation.

**Fig 5:**
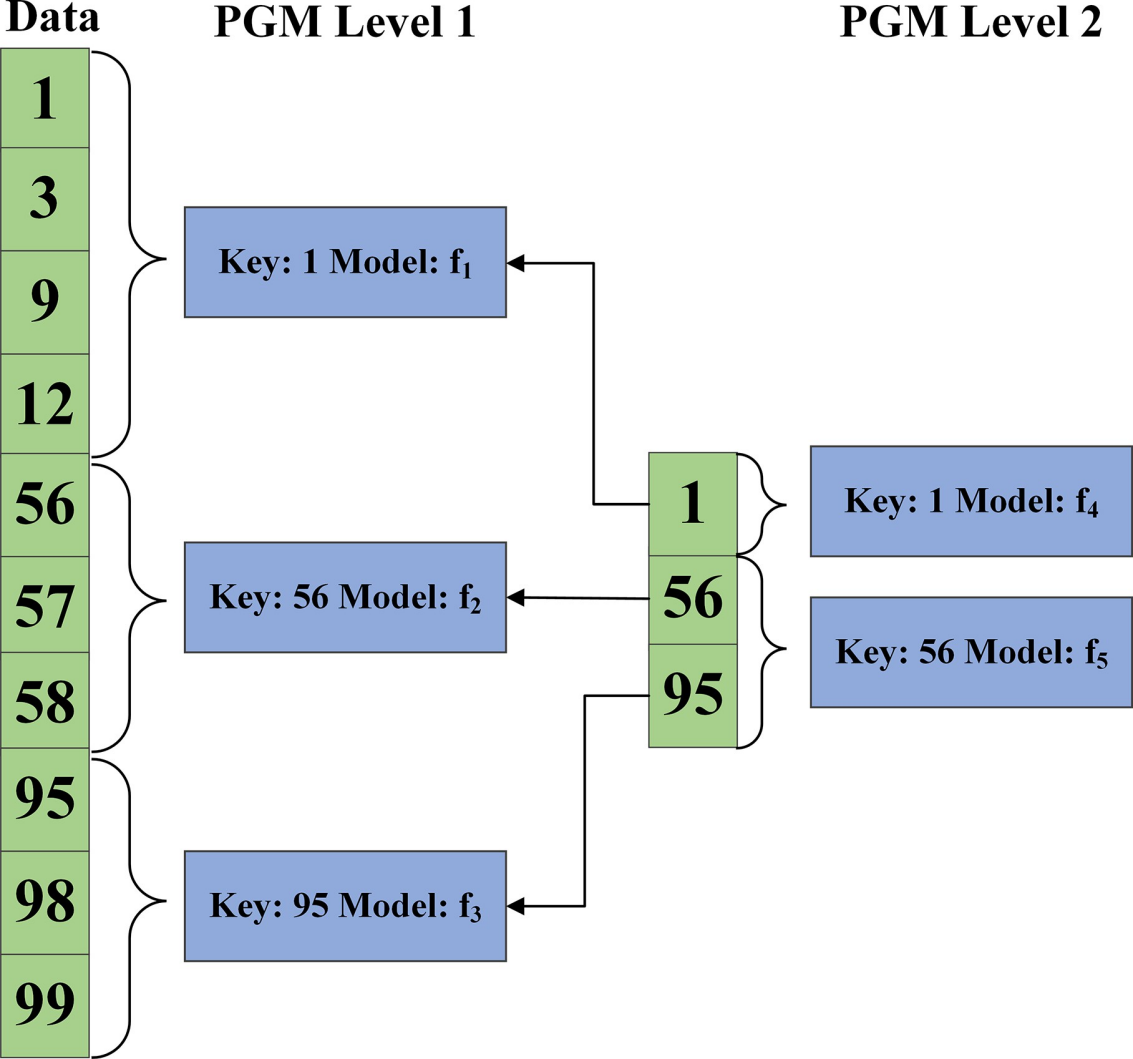
PGM index example diagram.

The use of a learning index on the NVM layer to predict the data position, thus enhancing the speed of data lookup. By index, the location of a key in *L*_0_ can be accurately determined. Firstly, each NVMTable is trained with a distinct index model. Secondly, an approximate mapping of the key to the data location is constructed. Then, starting from the approximate mapping and comparing the size of the mapping with the target key, the correct position is determined by searching linear search in the forward or backward direction. The Piecewise Geometric Model (PGM) index is a superior alternative. As shown in Fig 5, PGM index is a multi-level structure, where each level represents a segmented simple linear regression with different error rate lower bounds. The segmented linear regression divides the data into *n*+1 segments, which contain a set of points *p*_0_,*p*_1_,…,*p*_*n*_. It is represented as a segmented function, as shown in Eq (23).


$$F(x)=\{\begin{array}{l} a_{0}x+b_{0},x<p_{0}\\ a_{1}x+b_{1},x\geq p_{0}\wedge x<p_{1}\\ a_{2}x+b_{2},x\geq p_{1}\wedge x<p_{2}\\ \ldots \\ a_{n}x+b_{n},x\geq p_{n-1}\wedge x<p_{n} \end{array}$$ (23)

As shown in Fig 5, in the first layer, the data is divided into three segments, with each segment represented by a simple linear model (*f*_1_,*f*_2_,*f*_3_). As a result, each linear model is designed to predict the corresponding key values within its respective segments on the basis of predefined error values. Then, another error-bounded segmented linear regression is computed by treating the segmentation boundary of the first layer as its own sorted data set. This process is repeated until the PGM at the top level reaches an acceptable size. As for PGM index, it is constructed from the bottom to the top, which first picks an error bound and then reaches the bound with a minimal linear segmentation model. This step is repeated until the segmentation model is smaller than the threshold. Each regression treats a fixed error bound as an approximate index, and recur it. In the process, firstly constructing a segmented regression model using the underlying data, and then constructing another with fixed error using the splitting point of the first regression, where the Key lookup is a search process for each regression model until the underlying data is found.

## 4 On-chain enterprise composite blockchain construction

### 4.1 Conflux public blockchain construction

**Fig 6:**
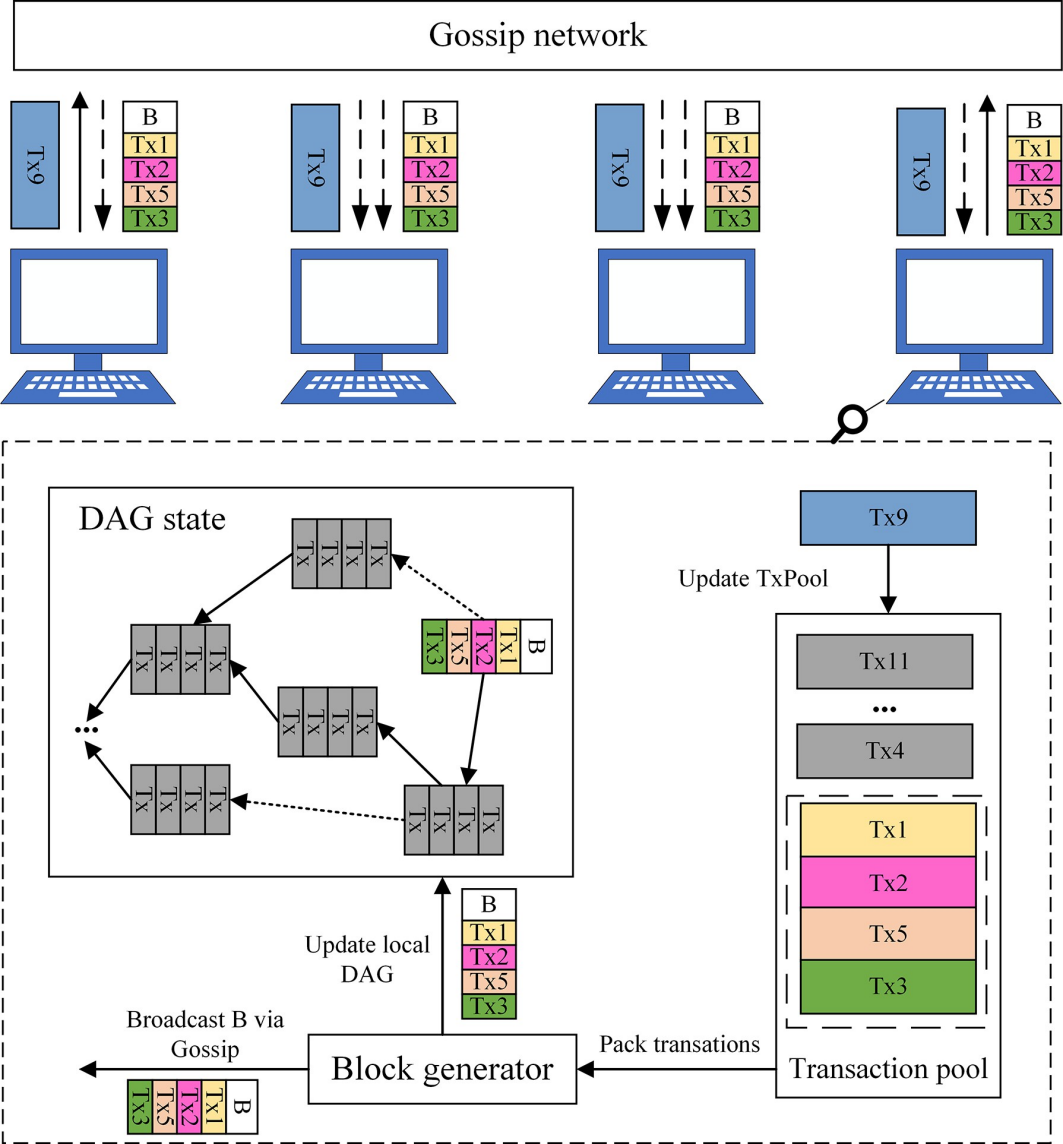
Conflux public blockchain architecture diagram.

The traditional public blockchain based on Bitcoin style exists deficiencies in scalability, throughput rate, and transaction confirmation delay, and so forth. As such, this paper adopts the DAG consensus protocol Conflux based on the backbone chain from the special structure of DAG to construct the Conflux public blockchain in the enterprise composite blockchain, and the architecture is shown in Fig 6.

In Conflux, the transactions consist of Key values corresponding to the enterprise raw data in the off-chain Level DB database as described in Section 3.2. Each transaction has been assigned a unique ID generated by the cryptographic digest function to ensure integrity. Additionally, a block consists of a list of transactions (four transactions in block B) and reference links to previous blocks (B links to the two previous blocks). Conflux starts with a predefined Genesis block to determine the initial state of the blockchain. All blocks and edges form a DAG, and potential variations in the contents of the DAG from node to node within a short period of time due to network latency, the ultimate goal of Conflux is to maintain consistency among nodes. In addition, all participating nodes are connected through the Gossip network, and whenever a node initiates a transaction, it will broadcast the transaction to all other nodes (Tx9). Similarly, when a node generates a new block, it also broadcasts the block to all other nodes (Block B).

In Conflux, each node maintains a pool of pending transactions, which covers the transactions that have been received by the node broadcast but have not yet been packed into any block. Upon receiving a new transaction, the node adds that transaction to its transaction pool (e.g., adding Tx9 to the transaction pool in Fig 6). Once a node generates a block or receives a block from another node, the node removes all transactions in the new block from its pending transaction pool. For example, in Fig 6, the node removes Tx1, Tx2, Tx3, and Tx5 after generating block B. Due to network latency and the existence of malicious nodes, concurrent blocks may pack duplicate or conflicting transactions, so it will be addressed by the consensus protocol. Each node runs a block generator to generate valid new blocks to pack pending transactions. Furthermore, each node maintains a local state containing all blocks perceived via broadcast. When a node discovers a new block, the node will update local DAG state accordingly.

**Fig 7:**
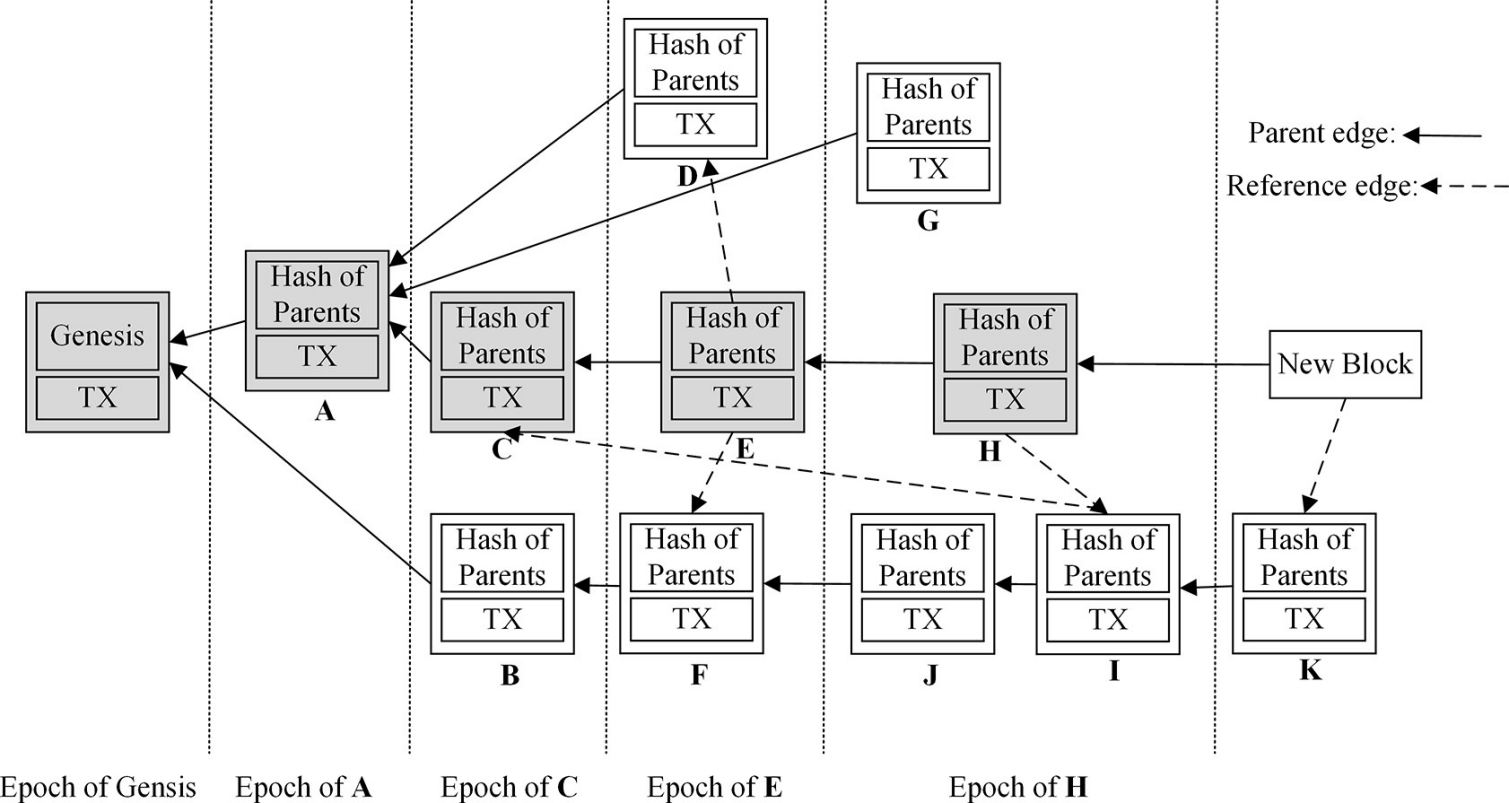
Conflux public blockchain data structure diagram.

The underlying block data structure of Conflux public blockchain is shown in Fig 7, which adopts the form of tree diagram to speed up the block-out speed by parallel processing, and does not compromise the system’s security due to the forking problem, thereby enabling each enterprise to upload transactions in parallel (the Hash value corresponding to the raw data of the enterprise). Ultimately, it allows the whole system to process blocks and transaction efficiently.

In Conflux public blockchain, a novel consensus mechanism is proposed, which can handle concurrent blocks without discarding any concurrent block as forks. In Conflux, except for the Genesis block, each block has one outgoing parent edge (solid arrow in Fig 7), which represents the voting relationship between blocks. Each block can have multiple outgoing reference edges (dashed arrows in Fig 7) that indicate the order of block generation. All parent edges together form a parental tree, where the Genesis block is the root. Conflux selects a chain from the Genesis block to one of the leaf blocks as the master chain. In the selection principle of the master chain in Conflux, it follows the Ghost protocol. This process begins at the Genesis block, and calculates the subtree size of each node. Generally, the largest subtree is as a node of the master chain. For example, in Fig 7, since A has more children than B, Conflux selects Genesis, A, C, E, and H as the master chain.

When a node generates a new block, it first computes the master chain in local DAG state and sets the last block in the master chain as the parent of the new block. Then, it identifies all end blocks without incoming edge and creates reference edges from the new block to each end block. In Fig 7, when a new block N is generated, the node selects H as the parent block of N and creates a reference edge from N to K. In the process of consensus, the sorting algorithm uses the master chain to divide all blocks into epochs, with each block on the master chain corresponding to an epoch. Blocks in each epoch can be reached from the corresponding block on the master chain through a combination of parent and reference edges, but does not include in previous epochs. In Fig 7, J belongs to the epoch H, because J can be reached from H, but not from any of the previous blocks of the master chain.

When performing consensus to determine the block total order, Conflux initially orders the blocks according to their corresponding epochs, followed by their topological order within each epoch. For the local DAG in Fig 7, Conflux obtains the following total order: Genesis, A, B, C, D, F, E, G, J, I, H, K. In addition, when ordering transactions, the order is determined by the block total order. If two transactions are in the same block, they are ordered by the appearance order. During the ordering process, if two transactions conflict with each other, Conflux will discard the second transaction. If a transaction appears in more than one block, Conflux will keep only the first transaction and discard the redundant transactions. By following the aforementioned process, data updating is performed on the Key value corresponding to the raw data uploaded by the enterprise.

### 4.2 Fabric consortium blockchain construction

**Fig 8:**
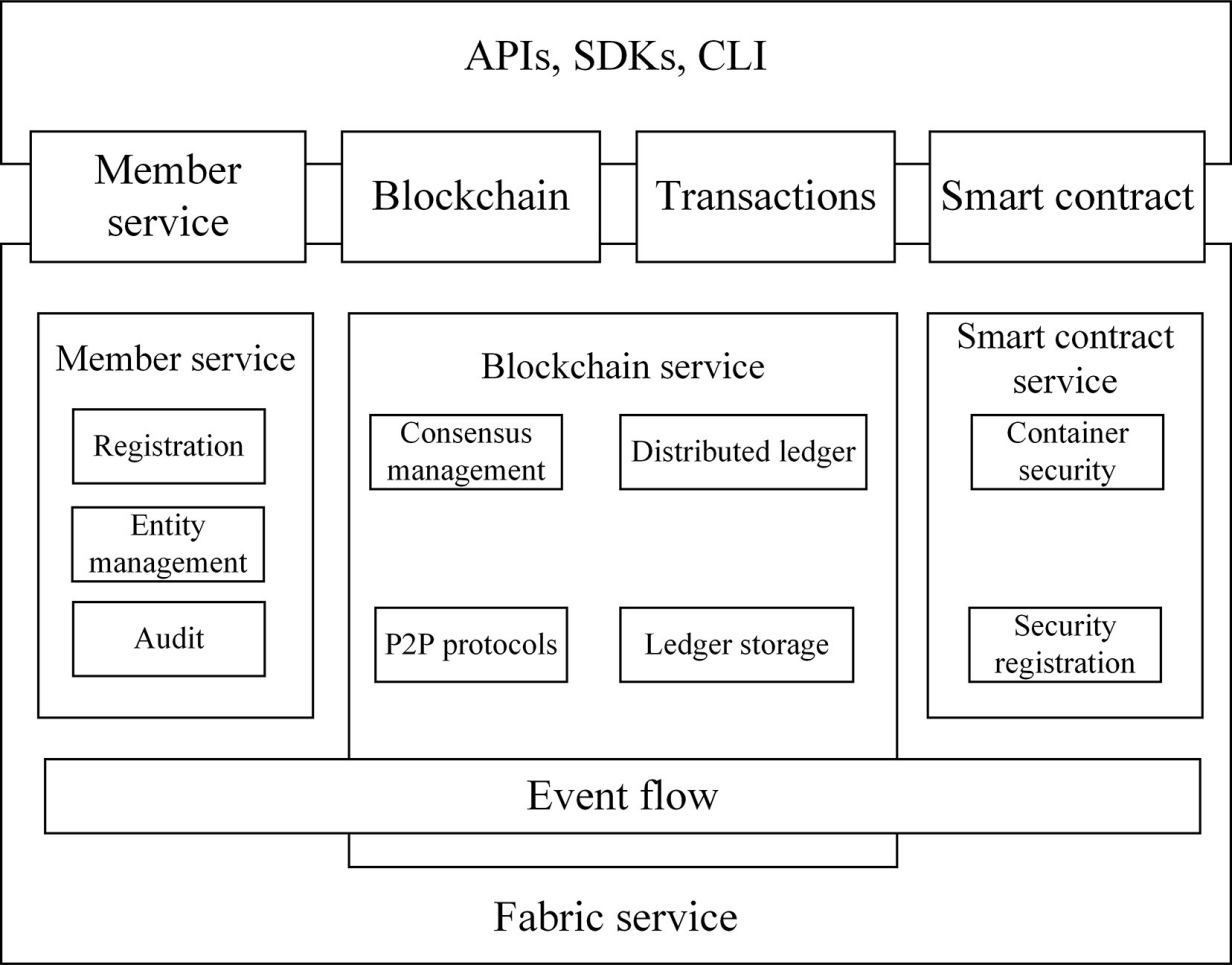
Hyperledger Fabric architecture diagram.

The consortium blockchain of the enterprise composite blockchain is responsible for storing the state data of enterprises from start-up, operation to end, facilitating data analysis for evaluating the enterprise business environment evaluation. Due to the large number of companies, this paper selects a certain number of pre-determined enterprise nodes to establish the consortium blockchain, and these nodes implement consensus algorithm, public verification, and secure data storage, thus reducing network resource consumption. To implement the consortium blockchain module, the Hyperledger Fabric framework, a well-known representative of the consortium blockchain, is used, and the Fabric architecture diagram is shown in Fig 8.

As illustrated in Fig 8, the Hyperledger Fabric architecture contains four primary components, namely member management service, blockchain service, smart contract and event flow [26], which are responsible for enterprise identity access identification, data storage sharing, business logic processing and asynchronous communication respectively. Among them, blockchain serves as the core architecture of Fabric platform, which facilitates services for communication between enterprise nodes by P2P protocol, distributed ledger between nodes, ledger storage and consensus mechanism management.

The consortium blockchain is designed to store enterprise state data, and the symbols used in stored procedures and their meanings are shown in Table 2. When the contract execution conditions are met are met, the smart contract is automatically triggered to accesses, shares and stores data. According to the pre-defined constraints, it performs data storage. The main workflow are as follows:

1. System initialization
  Initially, each enterprise node needs to undergo the identity authentication by the regulator node. Once the authentication is successful, the node becomes a valid participant in the consortium blockchain network; then it obtains ${\left\{PK_{PID_{i}^{k}},SK_{PID_{i}^{k}},Cert_{PID_{i}^{k}}\right\}}_{k=1}^{v}$, which represents the public-private key pair used for data encryption and certificate respectively; Finally, it performs the initialization of the system, where the enterprise node downloads the current block data storage location index table of consortium blockchain from the record pool of the neighboring enterprise headquarter nodes.
2. State data uploading
  Initially, enterprise node *N*_*i*_ initiates an upload request to local enterprise headquarter node *BS*_*j*_,which includes $Cert_{PID_{i}^{k}}$ and digital signature *Sig*_1 currently used by *N*_*i*_ to ensure the reliability and authenticity of the data source. Secondly, *BS*_*j*_ receives and verifies *N*_*i*_’s request and identity information to confirm legitimacy, and responds accordingly. Finally, *N*_*i*_ encrypts the state data *Data* using the public key $PK_{PID_{i}^{k}}$ in the current public-private key pair and sends this state data together with digital signature to *BS*_*j*_, and *BS*_*j*_ encrypts the record data sent by *N*_*i*_ through public key $PK_{BS_{j}}$ to obtain the final uploaded data *Record*. The formal description of the above process is as follows.
$$N_{i}\to BS_{j}:Record=E_{PK_{BS_{j}}}(Data\_1\|Cert_{PID_{i}^{k}}\|Sig\_1\|Timestamp)$$ (24)
  where $Data\_1=EPK_{PID_{i}^{k}}\left(Data\|Timestamp\right)$, $Sig\_1=Sig_{SK_{PID_{i}^{k}}}(Data\_1)$.
3. Uploading data collection
  *BS*_*j*_ validates the uploaded *Record*. If the data is secure and valid, it is stored in the local record pool, otherwise, it is ignored.
4. Proof of workload for local enterprise headquarter nodes
  After every period *T* (ten min in this paper), *BS*_*j*_ merges all valid data collected within *T* into a data set *Data*_*set*(Data_set={Records‖Timestamp}) with data signature to ensure the legitimacy and verifiability of source. *BS*_*j*_, who finds a valid workload proof first, gets the right to record this data block and is rewarded accordingly. The effective workload proof is that *BS*_*j*_ calculates the Hash of the current block based on the random number *x* and the values of *Hash*, *Timestamp*, Merkle tree root of the previous block *P*_*data*, where *x* needs to satisfy Hash(x+P_data)<Difficulty, and *Difficulty* is the difficulty value used to adjust the speed of *BS*_*j*_ to calculate the correct *x* value. *BS*_*j*_, which has obtained the bookkeeping right, needs to broadcast the current *Data*_*set* and the calculated *x* to other enterprise headquarter nodes for verification and validation. If pass, the *BS*_*j*_ merges the *Data*_*set* into a new data block and stores it in the consortium blockchain, and receives the corresponding system reward.
5. Block consensus among enterprise headquarter nodes

**Table 2:** Symbols and their meanings.

Symbols	Meanings
*N*_*i*_	Enterprise node *i*
*BS*_*j*_	Enterprise headquarter node *j*
*PK*_*i*_,*SK*_*i*_,*Cert*_*i*_	Entity *i*’s public key, private key and certificate
{*x*}	The set of element *x*
*Timestamp*	Timestamp
*i*→*j*	Entity *i* sends message to entity *j*
*x*‖*y*	Element *x* links element *y*
$E_{PK_{i}}(m)$	Encrypt message *m* using entity *i*’s public key
$Sign_{SK_{i}}(m)$	Digital signature of message *m* using private key of entity *i*
*Hash*(*m*)	The hash value of message *m*

*BS*_*j*_ becomes the master node (*Leader*) in the current consensus process, while other enterprise headquarter nodes are used as slave nodes (*Slave*), and the consortium blockchain adopts Practical Byzantine Fault Tolerance (PBFT) consensus mechanism for consensus, and the consensus process is as follows.

**Fig 9:**
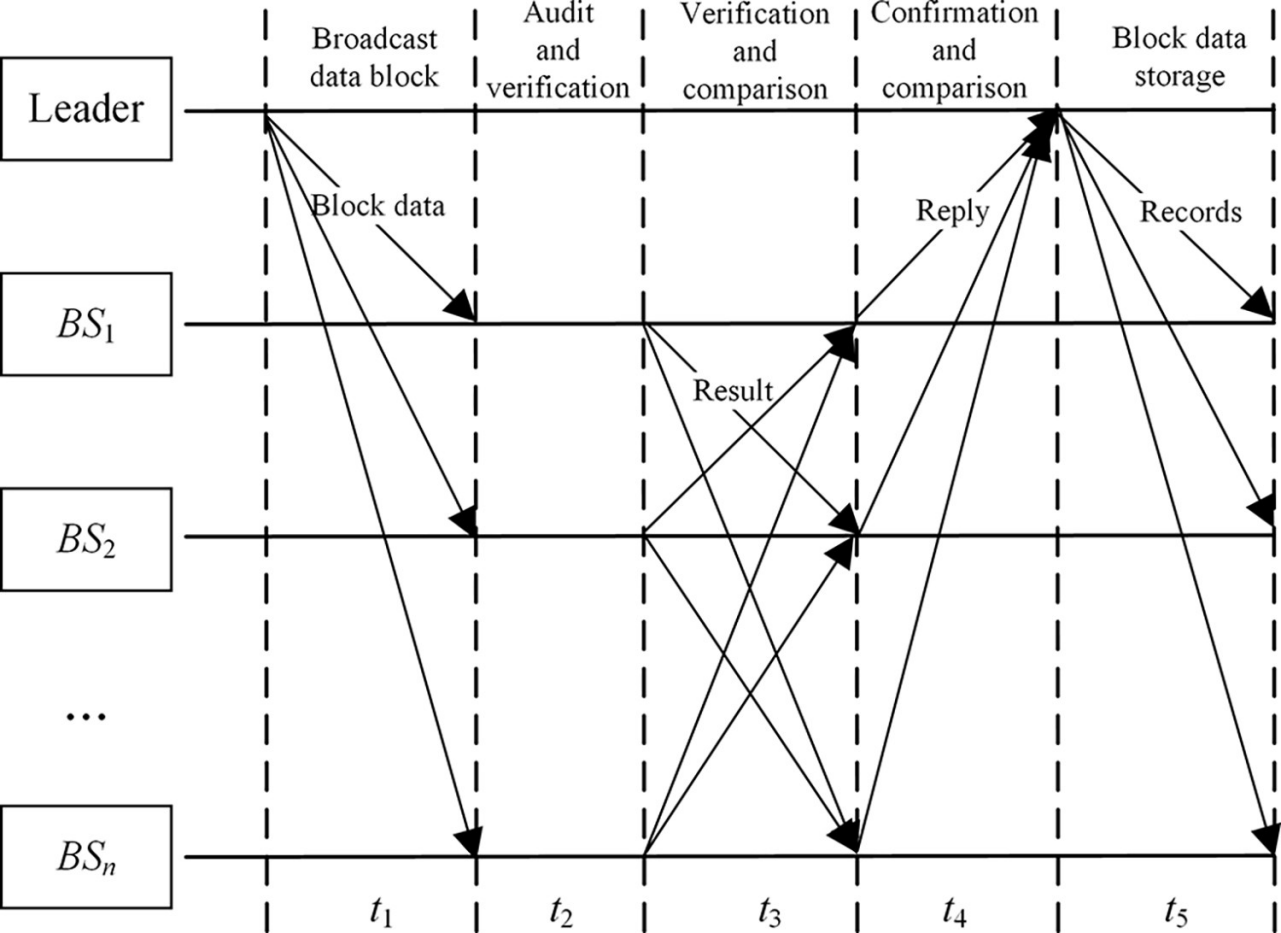
Consensus process for consortium blockchain.

1) Firstly, *Leader* collects the *Data*_*set* from *Slave* and consolidates it into a new data block with *Leader*’s digital signature and the *Hash* of the new data block appended for verification. Then, *Leader* broadcasts the new data block to each *Slave* to wait for verification by other nodes. The specific consensus process is shown in Fig 9, and the formal description of the above process is as follows.
$$BS_{j}\to All:Record=(Data\_sets\|Data\_hash\|Cert_{BS_{j}}\|Sig_{BS_{j}}\|Timestamp)$$ (25)
  where Data_hash=Hash(Data_sets‖Timestamp), $Sig_{BS_{j}}=Sign_{SK_{BS_{j}}}(Data\_sets\|Data\_hash)$.

2) Upon receiving the data block, *Slave* verifies the block *Hash* and digital signature and other information sent to confirm exactitude and legality, and broadcasts the verification result *Result* with own digital signature to other *Slave*, thus realizing interconnection and inter-checking among *Slave*s.
3) Certain slave node *BS*_*l*_ receives and aggregates the verification results of other *Slave*, then compares them with own verification result, and after verification, it sends a *Reply* to *Leader*, which includes *Slave*’s own verification results (*my*_*result*), all received verification results (*Rece*_*results*), the final conclusion of the verification comparison (*Comparision*), and the corresponding digital signature. The formal description of the aforesaid process is as follows.
$$BS_{l}\to BS_{j}:Reply=E_{PK_{BS_{j}}}(Data\_3\|Cert_{BS_{l}}\|Sig_{BS_{l}}\|Timestamp)$$ (26)
  where Data_3=my_result‖Rece_results‖Comparision, $Sig_{BS_{l}}=Sign_{SK_{BS_{l}}}(Data\_3)$.
4) *Leader* collects and aggregates the validation responses from all *Slave*. If all *Slave* verify the exactitude and legitimacy of the current block, *Leader* integrates the data block, the set of certificates of *Slave* involved in the verification ({*Cert*_*BS*_}) and the corresponding digital signatures, then sends them to all *Slave*. Thereafter, the data block will be stored in the consortium blockchain in chronological order, from which *Leader* also receives the system’s reward. The formal description of the above process is as follows.
$$BS_{j}\to All:Data\_block=(Data\_4\|Sig_{BS_{j}}\|Timestamp)$$ (27)
  where, $Data\_4=(Data\_sets\|Data\_hash\|\{Cert_{BS}\}\|Timestamp)$, $Sig_{BS_{j}}=Sign_{SK_{BS_{j}}}(Data\_4)$.
5) In case some enterprise headquarter nodes fail to pass the current verification results, *Leader* will analyze and check the verification results. If deemed necessary, the data block will be resent to these enterprise headquarter nodes for secondary validation, however if there still exist nodes that do not pass the validation, according to the principle of majority rule, only if more than a certain percentage of enterprise headquarter nodes have validated the data block, the data block will be loaded into the consortium blockchain through the process described in 4). Concurrently, *Leader* will further scrutinize the unverified results, and determine whether these nodes have malicious behaviors, and the malicious nodes will be promptly handled, so as to ensure the safe and stable operation of the system.

The aforementioned consensus process ensures that the enterprise state data will be stored in the consortium blockchain Fabric, so as to provide correlation data traceability and analysis support for the evaluation of enterprise business environment.

## 5 Experiments and analysis

### 5.1 Experimental setup

The experiments perform on the data set in XBlock, a blockchain data intelligence platform developed by InplusLab lab, a blockchain and smart finance research center. All blockchain datasets are standardized, cleaned and categorized, and unified into a standard format, and the specific contents of the datasets are shown in Table 3.

**Table 3:** Experimental datasets.

Dataset name	Description
First-order transaction network of phishing nodes	The network contains on average more than 60,000 nodes and 200,000 links
Bitcoin partial transaction dataset	Snapshots of transaction data from November 2014 to January 2016 are sampled with a 6-month interval. Each snapshot contains the first 1.5 million transaction records for the corresponding month
Second-order transaction network of phishing nodes	Contains 1660 targeted phishing nodes and 1700 transaction data generated by non-phishing nodes crawled from Etherscan
Ethereum on-chain data	Contains 14,500,000 block messages, 1,524,325,653 transactions generated from block data

The experiments were first conducted to validate the modified read and write performance in the off-chain Level DB. The modified Level DB is deployed in a standalone environment, and the experimental environment is shown in Table 4.

**Table 4:** Level DB read and write performance test environment.

Test environment	Parameters
Linux kernel version	x86_64 Linux 5.11.16-arch1-1
CPU	Intel Xeon Gold 6240R @ 96x 4 GHz model 48 cores 96 threads
DRAM	128 GB
NVM	512 GB
SSD	1 TB of NVMe interface

The experimental comparison of storage efficiency and storage overhead for on-chain enterprise composite blockchain storage was then performed. The experiment was carried out on a cluster of 20 nodes, where each node, equipped with a 32-core 2.10GHz Intel Xeon CPU, 128GB RAM, 10T storage space, was running CentOS version 7 operation system. Docker virtualization technology is utilized to deploy the blockchain, while Kubernetes is employed to manage docker clusters. The servers communicate with each other via a gigabit network and communicate between containers via flannel technology. All codes are written in C++, and Level DB is used to store off-chain data. The source codes are available at https://github.com/123lsiop/Enterprise-composite-blockchain-construction-method.

### 5.2 Comparison of read and write performance of Level DB data off the chain

Prior to modifying the design of *L*_0_, the reading operation latency mainly consists of reading memory and reading disk. The former includes the reading of MemTable and Immutable MemTable, while the latter is reading each layer of SSTable file from low to high. More specifically, reading disk comprises the following steps:

1. Reading the index in SSTable file to determine the location of data.
2. Copying the data block to the memory and deserializing it to memory format.
3. Finding the specific data in the data block.

Regarding the write operation latency, it encompasses three kinds of latency, one is incurred when writing the data and its checksum to disk before the data is written to the memory table. The second is the latency of writing data to MemTable, and the third is the tail latency brought by two merges (Minor Compaction and Major Compaction).

**Fig 10:**
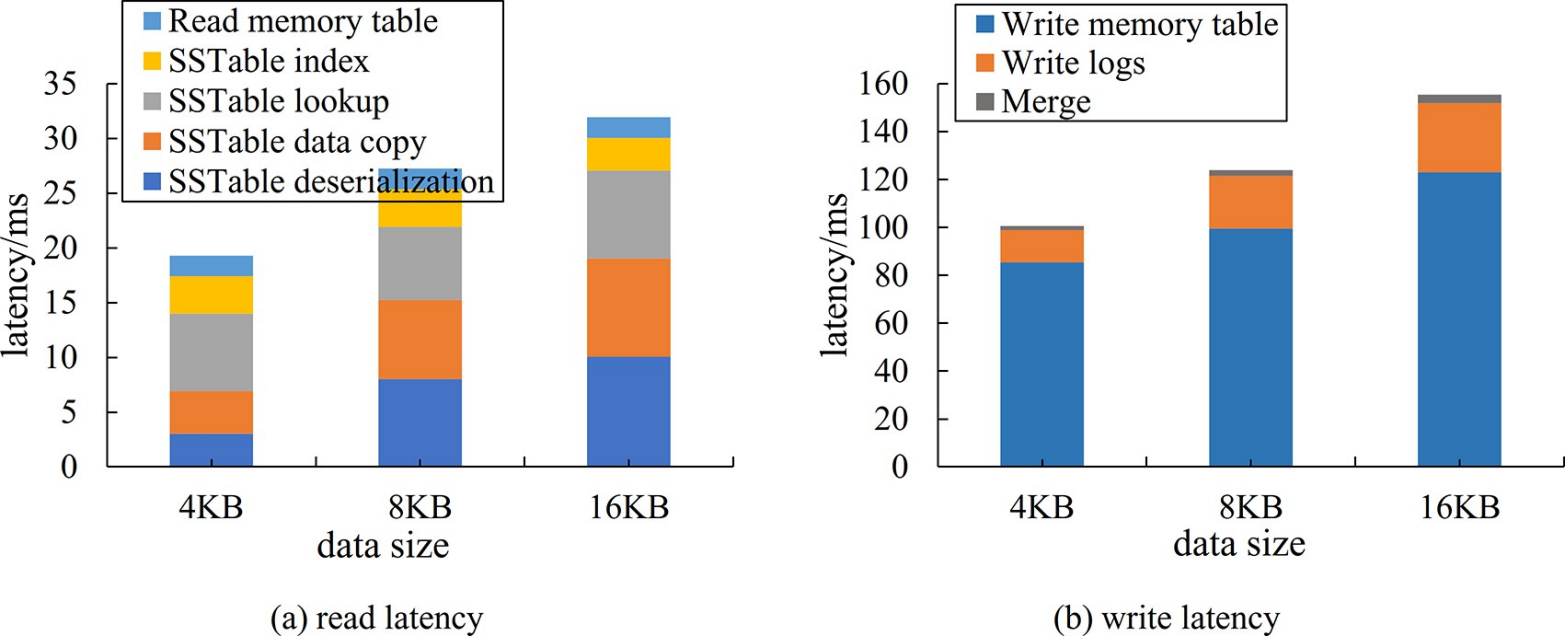
Comparison of read and write latency of Level DB before modification.

**Fig 11:**
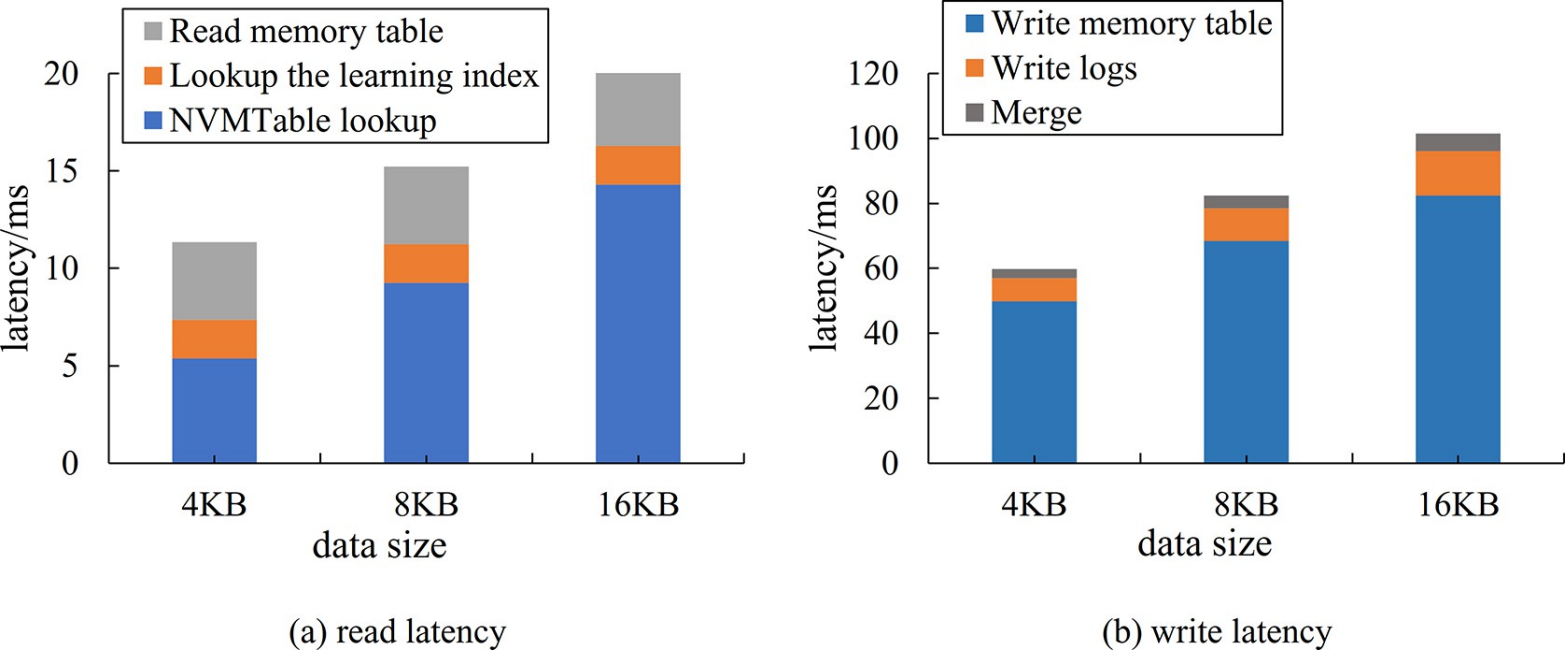
Comparison of read and write latency of Level DB after modification.

In the Level DB read and write performance test comparison, the upper limit number of *L*_0_ files is set to 10, and the latency is compared at three different sizes of 4kB, 8kB and 16kB Value. For each Value, 100,000 uniformly distributed test data are generated respectively, and then 100,000 queries uniformly distributed over the range of test data are generated. As shown in Figs 10 and 11, the read and write latency of Level DB before and after the modification are illustrated respectively.

As shown in Figs 10(A) and 11(A), on the one hand, the overall reading latency is reduced by about 50% by the modified *L*_0_ architecture. It can be attributed to placing the *L*_0_ layer on the NVM, which shortens the query path for the *L*_0_ layer. Additionally, CPU can directly access data on the NVM, as a result, the data copy and deserialization in Fig 10(A) are omitted. On the other hand, the lookup learning index is introduced, which is also more efficient than the binary search of the traditional SSTable.

As shown in Figs 10(B) and 11(B), the overall write latency is reduced by about 30% compared to both before and after the modified *L*_0_ layer design. Although the paths for writing memory tables and writing logs are basically unchanged, the process of memory table persistence is changed from DRAM to SSD to DRAM to NVM, the majority of the reduction in latency is attributed to the merge process.

### 5.3 On-chain enterprise composite blockchain storage efficiency comparison

**Fig 12:**
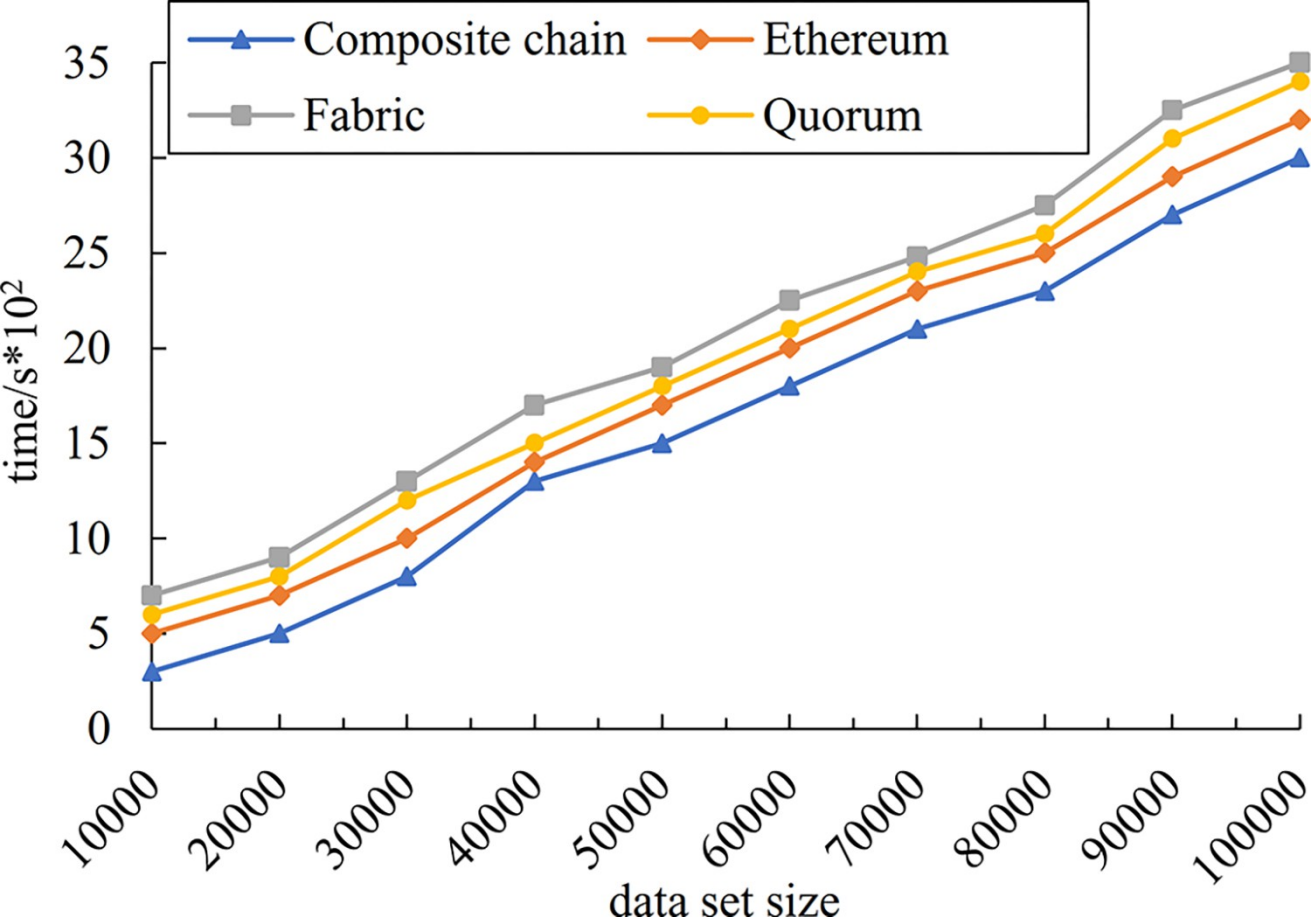
Enterprise composite blockchain storage efficiency comparison.

Experiment evaluated the storage efficiency of the enterprise composite blockchain compared to the public blockchain Ethereum and the enterprise blockchains Hyperledger Fabric and Quorum. As illustrated in Fig 12, the horizontal coordinate indicates the stored enterprise entity data set and the vertical coordinate indicates the time required for storage.

As can be seen in Fig 12, the storage efficiency of the enterprise composite blockchain storage structure proposed in this paper is faster than others. It is because that the public blockchain in the composite blockchain adopts the DAG-based block structure and Conflux consensus. For one thing, the DAG-based block structure adopts the form of tree diagram, which speeds up the block-out speed by parallel processing and does not reduce its security due to the forking problem. For another thing, Conflux consensus adopts Ghost protocol to select the master chain and selects the largest subtree as a node of the master chain, so that every enterprise can upload transactions in parallel, which accelerates the block construction and consensus and ultimately enhances the storage efficiency.

### 5.4 On-chain enterprise composite blockchain storage overhead comparison

**Fig 13:**
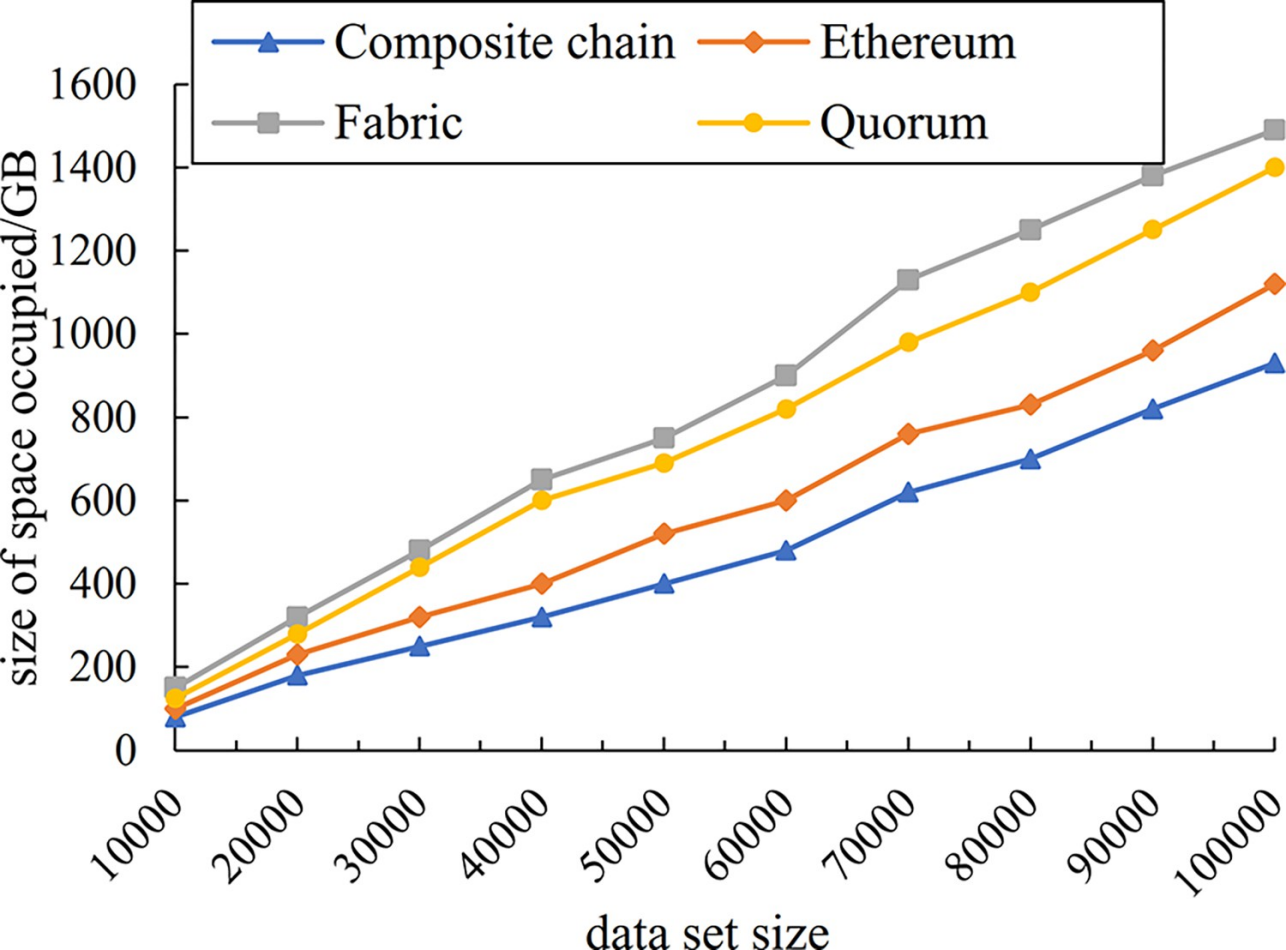
Enterprise composite blockchain storage overhead comparison.

The experiment simulated the overhead of blockchain storage structure using enterprise composite blockchain compared with that using the public blockchain Ethereum and the enterprise blockchains Hyperledger Fabric and Quorum. The horizontal coordinate indicates the storage enterprise entity data set and the vertical coordinate indicates the size of space required for storage, and the experimental results on overhead are presented in Fig 13.

As can be seen from Fig 13, the storage overhead of the proposed enterprise composite blockchain storage structure is lower than that of Ethereum, Fabric and Quorum. The reduction in storage overhead is more pronounced as the volume of data increases, as the enterprise composite blockchain employs a hybrid on-chain and off-chain storage, while the consortium blockchain serves as an auxiliary storage for enterprise state data and can provide indexing support for the public blockchain, which thus further reduces the communication and storage burden of the blockchain system. Consequently, this approach effectively alleviates the problem of high pressure on-chain storage.

## 6 Conclusion

The business environment plays a critical role in the business condition of enterprises and local economic development. However, the evaluation of business environment is often hindered by the low quality of enterprise raw data. To address this issue, an enterprise composite blockchain construction method is proposed for business environment, which leverages a hybrid on-chain and off-chain storage mode. Specifically, the improved hash function SHA256 algorithm is introduced to encrypt the enterprise raw data and store it in the off-chain Level DB database based on non-volatile memory. Then, the data are stored on chain, and the Key values in Level DB are stored to the DAG-based Conflux public blockchain, and the enterprise state data are deposited to the consortium blockchain Hyperledger Fabric, correspondingly to provide credible deposition data for business environment evaluation respectively. Finally, experimental comparison and analysis verified the effectiveness of the proposed method.

Indeed, the work presented in this paper represents a significant step towards improving the performance and reliability of blockchain systems for data storage in business environment evaluation. However, there are several unresolved research questions that require further investigation. For example, future research could focus on investigating the potential of blockchain-based decentralized identity systems and their impact on privacy, security, and user control on private enterprise data. Additionally, the impact of emerging consensus algorithms and governance models on the scalability, efficiency and decentralization of blockchain networks could also be analyzed.
